# Changes in practice patterns affecting in-hospital and post-discharge survival among ACS patients

**DOI:** 10.1186/1472-6963-6-140

**Published:** 2006-10-24

**Authors:** Manfred Stommel, Ade Olomu, Margaret Holmes-Rovner, William Corser, Joseph C Gardiner

**Affiliations:** 1 College of Nursing, Michigan State University, East Lansing, Michigan, USA; 2 Department of Internal Medicine, College of Human Medicine, Michigan State University, East Lansing, Michigan, USA; 3 Center for Ethics, College of Human Medicine, Michigan State University, East Lansing, Michigan, USA; 4 Department of Epidemiology, Michigan State University, East Lansing, Michigan, USA

## Abstract

**Background:**

Adherence to clinical practice guidelines for the treatment of specific illnesses may result in unexpected outcomes, given that multiple therapies must often be given to patients with diverse medical conditions. Yet, few studies have presented empirical evidence that quality improvement (QI) programs both change practice by improving adherence to guidelines and improve patient outcomes under the conditions of actual practice. Thus, we focus on patient survival, following hospitalization for acute coronary syndrome in three successive patient cohorts from the same community hospitals, with a quality improvement intervention occurring between cohorts two and three.

**Methods:**

This study is a comparison of three historical cohorts of Acute Coronary Syndrome (ACS) patients in the same five community hospitals in 1994–5, 1997, 2002–3. A quality improvement project, the Guidelines Applied to Practice (GAP), was implemented in these hospitals in 2001.

Study participants were recruited from community hospitals located in two Michigan communities during three separate time periods. The cohorts comprise (1) patients enrolled between December 1993 and April 1995 (N = 814), (2) patients enrolled between February 1997 and September 1997 (N = 452), and (3) patients enrolled between January 14, 2002 and April 13, 2003 (N = 710).

Mortality data were obtained from Michigan's Bureau of Vital Statistics for all three patient cohorts. Predictor variables, obtained from medical record reviews, included demographic information, indicators of disease severity (ejection fraction), co-morbid conditions, hospital treatment information concerning most invasive procedures and the use of ace-inhibitors, beta-blockers and aspirin in the hospital and as discharge recommendations.

**Results:**

Adjusted in-hospital mortality showed a marked improvement with a HR = 0.16 (p < 0.001) comparing 2003 patients in the same hospitals to those 10 years earlier. Large gains in the in-hospital mortality were maintained based on 1-year mortality rates after hospital discharge.

**Conclusion:**

Changes in practice patterns that follow recommended guidelines can significantly improve care for ACS patients. In-hospital mortality gains were maintained in the year following discharge.

## Background

While clinical practice guidelines are usually developed based on strong evidence from randomized clinical trials (RCTs), their consistent application in clinical practice remains challenging. RCTs are normally designed to examine only one or two therapies at a time and often enroll patients with fewer co-morbid conditions, who are at overall lower risk for mortality. In the 'real world' of clinical practice, multiple therapies must often be given all at once and to patients, for whom the expected benefit may be less certain, such as the elderly. Adhering to the guidelines may therefore result in unexpected outcomes when applied in community hospital settings. Thus, it is important to show that quality improvement (QI) programs do not only change practice by improving adherence to guidelines for hospital care [1-3], but they must also be shown to improve patient outcomes in both short and longer terms.

In this paper, we examine how a decade of changes and improvement in hospital care for ACS patients have affected both in-hospital and one-year post-hospital mortality. In particular, we evaluate treatment changes and mortality outcomes in three ACS patient cohorts observed over 10 years in the same five community hospitals, which, during the final cohort observations, participated in a quality improvement (QI) program.

## Methods

### Design

The study compared the survival of ACS patients in three historical cohorts enrolled at the same five community hospitals during 1994–5, 1997, and 2002–3. A QI project, the Guidelines Applied to Practice (GAP) project was implemented in these hospitals in 2001 [1], one year prior to the data collection for the latest cohort.

### Setting and participants

The five community-hospitals were located in two geographically contiguous Michigan communities. These communities have comparable patient demographics, employment patterns, insurance coverage, proportions of minority, unemployed and low-income residents [4-6], and their characteristics have remained quite stable over the 10-year period under consideration [7].

The earlier two patient cohorts were identified in the Michigan Inter-institutional Collaborative Heart (MICH) study [8]. This study was an ongoing assessment of ACS practice patterns and patient outcomes in the two mid-Michigan communities. In Phase I, 828 consenting patients were admitted with ACS to one of the five area hospitals between December 1993 and April 1995, with complete record audits available for 814. Phase II enrolled 502 consenting ACS patients admitted between February 1997 and September 1997; of these, 452 had complete medical records. While the definition of ACS is a recent change from the earlier definition of AMI, the eligibility criteria (positive enzymes, a working diagnosis of AMI or ACS and symptoms) were the same for all three cohorts, with the exception that the enzyme used was CPK-mb in the first two cohorts and Troponin in the third. The "normal" vs. elevated cut points at each of the admitting hospitals were used in all cases.

The third cohort came from the HARP study ("Heart After Hospital Recovery Planner"), a behavioral intervention study designed to evaluate the effectiveness of telephone interventions to improve post-discharge cardiovascular health behavior in patients hospitalized for ACS. This study was conducted in the same hospitals as the MICH studies, with patients enrolled between January 14, 2002 and April 13, 2003.

The five hospitals were also sites for the American College of Cardiology (ACC) AMI GAP QI initiative, which took place in 2001. Designed to improve the quality of care according to the ACC/American Heart Association (AHA) Practice guidelines, the GAP initiative was a multifaceted intervention consisting of a project kick-off presentation, creation and implementation of a customized tool kit which included standardized: a) AMI medication and treatment order sheets, b) clinical pathways to coordinate in-hospital care, and c) discharge medication and referral forms based on ACC/AHA guidelines. Details of the intervention have been described elsewhere [1].

For the HARP-cum-GAP cohort, medical record audits could be obtained from all 719 ACS patients, who consented to being in the study. While patients in the HARP study were randomized to a telephone behavioral intervention after hospital discharge, mortality rates between intervention and control groups did not differ. For the current analysis, all 710 HARP patients with complete records were included, after separate analyses involving only the HARP control group subjects did not show any difference in observed outcomes or patterns of predictors.

All three studies (MICH I, MICH II, HARP) were approved by the University Committee on Research Involving Human Subjects (UCRIHS) of Michigan State University and by each of the participating Hospital Institutional Review Boards before data were collected.

### Measures

For all three cohorts, mortality information, extending to at least one year of follow-up after the index hospitalization, was obtained from the Michigan Bureau of Vital Statistics. Predictor variables included demographic information, such as age (in years), gender (female vs. male), minority status (minority vs. non-Hispanic whites); type of health insurance (private/commercial, Medicare, Medicaid, none); indicators of the severity of the disease (ejection fraction, number of diseased vessels) and co-morbid conditions [9]; and in-hospital treatment information concerning the invasive procedure and treatments performed: Coronary Artery Bypass Graft Surgery (CABG), Percutaneous Transluminal Angioplasty (PCI), or Cardiac Catheterization (CATH) only. Finally, we also obtained information on the in-hospital use of ace-inhibitors, beta-blockers and aspirin as well as the discharge recommendations concerning these medications.

### Analysis

Initially, we generated descriptive statistics (means, medians and percentages) to compare the three study cohorts in terms of the demographic and clinical characteristics of patients. Differences between the groups were assessed using χ^2 ^tests for categorical variables and *t *tests (or Wilcoxon rank-sum tests) for continuous variables, as appropriate. In addition, unadjusted survival curves were generated using Kaplan-Meier methods [10]. Survival times were measured twice: (1) At first, we examined survival in the hospital, counting survival days from the date of admission to the index hospital to either the date of death or the date of discharge; (2) then we examined post hospital survival from the day of discharge to either the date of death or the censor date of 365 days after the index hospital admission.

For the multivariate analysis, we employed Cox proportional hazard regressions [10]. The adequacy of the proportional hazard assumptions was assessed, using graphical methods and the test by Grambsch & Thernau based on Schoenfeld residuals [11]. The relative strength of a variable's predictive contribution was estimated using the chi-square difference statistic, while the models' discrimination abilities were assessed using the C-statistic [12]. All analyses were carried out with STATA 9.2 software.

## Results

The demographic characteristics of the three cohorts are quite similar, even though contrasts reveal that the average age of patients in the HARP cohort is about 3–5 years lower than in the MICH cohorts (Table 1). There were greater differences with respect to the severity of illness. Members of the HARP cohort generally appear somewhat healthier, with fewer co-morbid conditions. They also included relatively few patients with low (<35%) ejection fractions, if only valid data are included: Among HARP patients, 12.3% (out of the 612 valid responses) had low ejection fractions, compared to 13.8% (out of 600) in MICH 1 and 18.3% (out of 235) in MICH 2. Likewise, the average number of diseased coronary vessels is somewhat lower in the HARP cohort. Concerning invasive diagnostic procedures and treatments, the main change in these five hospitals appears to have been a substantial increase in invasive treatments with PCI, from a low of 26.5% among MICH 1patients to 48.2% of all patients so treated in the most recent (HARP) cohort. Similar increases were observed for medications administered in the hospital and recommended upon discharge: between 1994 and 2003, the use of Angiotensin Converting Enzyme Inhibitors (ACEIs) and Angiotensin Receptor Blockers (ARBs), β-blockers and Aspirin increased substantially, both in the hospital and after discharge. By contrast, use of thrombolytic therapy fell as use of PCI increased.

**Figure 1:**
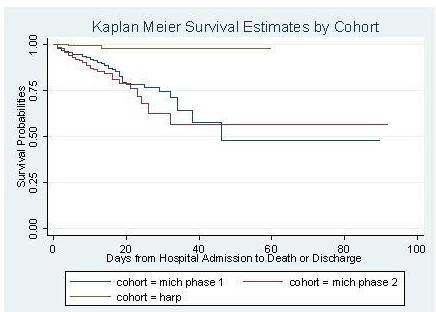
Unadjusted Survival Curves Comparing Three Patient Cohorts from the Same Hospitals during Index Hospitalization.

Figure 1 shows the unadjusted in-hospital survival of the ACS patients after admission. The mean hospital stay in the HARP cohort was substantially shorter than for the two MICH cohorts (10.7 for MICH1, 9.4 for MICH2 and 6.6 for HARP; F(2,1973) = 31.1, p < 0.01). Survival rates were lower in the earlier MICH cohorts (Mantel-Haenszel's Log-rank Test: χ^2 ^= 48.02; p < 0.001) compared to the more recent HARP cohort, while survival differences between the two MICH cohorts did not reach statistical significance.

Table 2 presents the adjusted survival rates. All predictor variables in the model meet the proportionality test for in-hospital survival. The results indicate a substantial rise in mortality hazards for patients with more than one co-morbid condition – the Charlson index includes heart disease, thus all subjects in this study have one 'co-morbidity', but the logarithmic shape of the fitted line implies that the impact of *additional *co-morbid conditions lessens with the presence of more co-morbid conditions. ACS patients with ejection fractions of 45% or higher had a mortality hazard only 38% as large as those for patients with ejection fractions below 35% (the contrast to the 35–44% category is also significant – p < 0.03). Invasive procedures were associated with substantial reductions in mortality, with hazard ratios of less than 42% compared to patients who did not undergo invasive procedures (none of the contrasts among the three listed procedures is significant). Among the medication categories (ACEIs and ARBs, aspirin and thrombolytics), three show hazard ratios whose expected magnitudes were associated with a risk reduction in mortality of between 44 and 57% (p < 0.005).

Interaction effects between procedures and the use of medications were generally non-significant, providing no evidence for cumulative procedure-medication benefits. However, the interaction between a dichotomized age variable (<65 vs. 65+) and a dichotomized procedure variable (no invasive procedure vs. having an invasive procedure) suggested that invasive procedures were more beneficial among younger (<65) ACS patients. Among younger patients, the overall mortality risk associated with invasive procedures was only 12% of the risk among other young patients, who did not get invasive procedures (HR = 0.12, p < 0.001). By contrast, among older patients the mortality risk was reduced to approximately 43% after an invasive procedure (HR = 0.43, p < 0.001).

**Figure 2:**
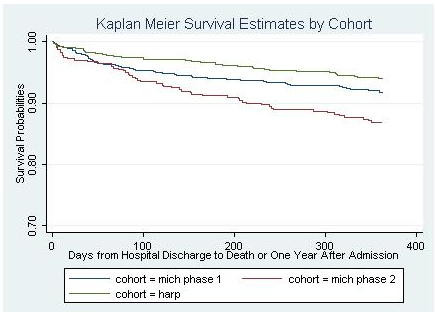
Unadjusted Survival Curves Comparing Three Patient Cohorts from the Same Hospitals Starting with Hospital Discharge to One Year After Hospital Admission.

Figure 2 shows the unadjusted mortality risks of the ACS patients after their discharge form the index hospital until one year after the initial heart event. As the Log-Rank Test confirms, survival rates differed significantly among the three cohorts (χ^2 ^= 17.55; p < 0.001), but post-hoc comparisons revealed that only the MICH 2 cohort had a significantly lower unadjusted survival rate than the other two cohorts.

Table 3 presents the adjusted hazard ratios. Originally, we tested a hazard model that contained both in-hospital medication use and discharge medications. However, due to substantial co-linearity among the hospital and discharge medications, we only retained the discharge medications in the model presented. Again, all predictor variables in the model meet the proportionality test for post-hospital survival. The results indicate the continued impact of co-morbid conditions and impact of invasive procedures on post-discharge survival. However, age is now a predictor of mortality, with the risk of mortality increasing by 3% for each additional year of age. Among the discharge medications, the use of beta-blockers reduces the post-hospital mortality risk almost by half, while aspirin use reduces it by a third. Finally, the higher mortality risks among MICH 2 patients disappear, after accounting for the fact that patients in this cohort had the highest average age and were least likely to undergo invasive procedures. Thus, no cohort differences in mortality risks remain, with the largest effect attributable to differences in invasive procedures (see chi-square difference values).

We also examined overall one-year mortality among all patients (138 died in the hospital and 152 after discharge from the hospital), using a Cox proportional hazard model; but this time, the base hazard was stratified to account for non-proportionality in the various medication groups. Again, due to co-linearity among in-hospital and post discharge medication use, we examined two separate models with either hospital or discharge medications as predictors. (The latter proved slightly stronger predictors of survival). Concerning the cohort comparison, overall one-year mortality risks were significantly larger in the MICH 2 cohort (HR = 1.48, p < 0.001), compared to the MICH 1 cohort, and were significantly smaller in the HARP cohort (HR = 0.30, p < 0.001). In short, the differentials in mortality risks, and especially the improvements apparent in the HARP/GAP cohort, all accrued during the hospital stay. After their hospital discharge, patients in the more recent HARP/GAP cohort did not experience additional incremental survival benefits, but, at the same time, mortality gains in the hospital were maintained at 1-year follow-up.

## Discussion

During recent decades, mortality attributable to coronary heart disease has declined, particularly among men [13]. These declines have been driven by improved early access to emergency medical care, but also by changes in treatment during hospitalization and after discharge [1,14]. Recent clinical trials testing the management of AMI and ACS have demonstrated efficacy of invasive hospital management [15-18]. Cumulative meta-analysis of randomized trials in myocardial infarction has revealed that long-term administration of beta-blockers leads to reductions in cardiac deaths, re-infarction rates, and risks of sudden death [19]. Similarly, benefits from the appropriate use of aspirin, lipid lowering agents, and ACEIs have been proven in multi-center, randomized, double-blind, placebo-control trials [20-23]. Several large-scale observational studies have also demonstrated a decreased mortality from adherence to published guidelines, which emphasize the systematic use of medications such as ACEIs, ARBs, or β-blockers for all eligible patients following AMI or ACS [24-26].

During the 1980s and 1990s, prior to the more recent trials, practice patterns showed increased use of thrombolytic therapy and PCI, and decreased use of CABG in many geographic areas [27-31]. In the past decade, practice guidelines have emphasized the use of Aspirin, β-blockers and ACEIs during and after hospital discharge for ACS [1]. However, the quality of care for ACS has remained less than optimal in many settings, and recent studies have continued to show a lack of adherence to published guidelines, though improvements have been noted [1,14,32,33]. Recently, the National Study of Physician Awareness and Adherence to Cardiovascular Disease Prevention Guidelines in 2005 revealed that only 40–50% of primary care physicians and cardiologists incorporated guidelines into clinical practice [34].

Evaluation of changes in practice resulting from exposure to QI interventions is complicated by the fact that mortality and other outcomes vary widely depending on patients' risk profile [35]. Clinical characteristics, such as co-morbid conditions and ejection fraction, substantially influence post-AMI and ACS survival. While some clinicians are reluctant to treat frail patients aggressively, invasive procedures for sicker patients with more co-morbid conditions and lower ejection fractions have often been advocated [28,36-38]. Finally, there is substantial evidence that socio-demographic characteristics such as increased age, minority race or ethnicity, and low income/low socio-economic status are predictors of in- and post-hospital mortality [4,38,39].

The evidence presented here is based on the comparison of historical cohorts. While such comparisons are, in principle, vulnerable to changes in population characteristics as well as changes in technology increasing the probability of incommensurate assessments, we believe that the current comparison sheds light on how changing practice patterns can have large effects on clinical outcomes. Since the comparisons were based on ACS patients presenting in the same hospitals in communities, whose populations remained stable over the last decade with little in- and out-migration, and since all three patient cohorts had to meet very similar eligibility criteria, the threat of selection bias appears small. In addition, we controlled statistically for demographic indicators (age, gender, race) as well as indicators of the disease severity (ejection fractions) and co-morbid conditions. However, we do acknowledge that information about ST elevation was not obtained in all cohorts and could not be used as a control variable.

## Conclusion

The finding that the most recent cohort (HARP/GAP) had substantially reduced hospital mortality is highly encouraging particularly in light of the fact that post-discharge mortality benefits were maintained, even when considering that many of the sicker patients survived their hospital stay in the later cohort. For example, 24.6% and 22.3% of the patients with 4 or more co-morbid conditions in the MICH 1 and MICH 2 cohorts respectively died during their hospital stay, while only 2.4% of the patients in HARP/GAP with 4 or more co-morbid conditions died in the hospital. Similarly, while 14.4% (17.8%) of the MICH 1 (MICH 2) patients over 64 years of age died in the hospital, that rate was cut to 0.7% among the older patients of the HARP/GAP cohort. While progress has also been made among the younger and healthier ACS patients, the advances among older and sicker patients are particularly impressive. This analysis shows that the in-hospital mortality improvements were sustained in the first year post-hospitalization, despite shortened length of stay. It appears that a quality improvement initiative has shown substantial improvement in survival for ACS patients attributable to increased use of effective treatments, while decreasing length of hospital stay.

## Competing interests

The author(s) declare that they have no competing interests.

## Authors' contributions

MS was primary author and statistical analyst. AO wrote and revised sections of the paper and provided medical expertise. MHR designed the study, contributed to the conceptualization of the paper and wrote sections of the paper. WC provided information for and wrote a section of the paper. JCG provided analytical advice and contributed to an early version of the paper. All authors read and approved the final manuscript.

## Pre-publication history

The pre-publication history for this paper can be accessed here:



## Figures and Tables

**Table 1:** Patient Characteristics and Treatments in Three Cohorts (n = 1,976)

Variables:	MICH1 n = 814	MICH2 n = 452	HARP n = 710	p-value
Demographics:				
Age	M = 63.8	M = 65.6	M = 60.7	<0.01
	SD = 13.7	SD = 14.5	SD = 12.7	
Gender (male)	510 (62.7%)	266 (58.9%)	443 (62.4%)	>0.36
Race (white)	682 (83.8%)	364 (80.5%)	606 (85.4%)	>0.09
				
Charlson Index	M = 2.32	M = 2.69	M = 2.09	<0.01
Med = 2	Med = 2	Med = 2		
	Range = 1–10	Range = 1–12	Range = 1–12	
				
Ejection Fraction				<0.01
<35%	83 (10.2%)	43 (9.5%)	75 (10.4%)	
35%–44%	121 (14.9%)	46 (10.2%)	115 (16.0%)	
≥45%	396 (48.7%)	146 (32.3%)	422 (58.7%)	
missing	214 (26.3%)	217 (48.0%)	107 (14.9%)	
				
No. of Vessels				<0.01
0	28 (3.5)	14 (3.3)	66 (11.0)	
1	196 (24.6)	84 (20.0)	207 (34.3)	
2	180 (22.6)	80 (19.0)	155 (25.7)	
3	392 (49.2)	243 (57.7)	175 (29.0)	
Invasive Procedures and Treatments:				
None	200 (24.6)	142 (31.4)	72 (10.5)	<0.01
CATH	614 (75.4)	312 (68.4)	638 (89.5)	<0.01
PCI	216 (26.5)	136 (30.1)	342 (48.2)	<0.01
CABG	185 (22.7)	74 (16.4)	158 (22.3)	<0.01
				
Medications in Hospital:				
ACEIs & ARBs	161 (19.8)	154 (34.7)	407 (57.3)	<0.01
Beta-Blockers	186 (24.1)	186 (41.2)	500 (70.4)	<0.01
Aspirin	498 (61.2)	307 (67.9)	614 (96.5)	<0.01
Thrombolytics	480 (59.0)	278 (61.5)	77 (10.9)	<0.01
				
Medications at Hospital Discharge:				
ACEIs & ARBs	161 (19.8)	154 (34.7)	407 (57.3)	<0.01
Beta-Blockers	186 (24.1)	186 (41.2)	500 (70.4)	<0.01
Aspirin	498 (61.2)	307 (67.9)	614 (96.5)	<0.01

**Table 2:** Mortality Hazard During Stay at Index Hospital by Patient Characteristics, Treatments and Time Cohort (n = 1,972)

**Variables:**	**Hazard Ratios**	**95% CI**	**p-value**	**Δχ**^**2**^**-value**
Age	1.01	0.99–1.02	>0.28	1.16
Gender (male)*	0.78	0.55–1.10	>0.16	2.01
Race (white)*	1.16	0.73–1.87	>0.53	0.40
Charlson Index***	2.03	1.43–2.88	<0.01	15.69
Ejection Fraction (<35%)*				8.71**
35%–44%	0.96	0.46–2.03	>0.92	0.01
≥45%	0.38	0.18–0.82	<0.01	6.15
missing	1.00	0.57–1.77	>0.99	<0.01
Invasive Procedures (none)*				23.01**
CATH	0.31	0.17–0.58	<0.01	13.56
PCI	0.42	0.23–0.79	<0.01	7.21
CABG	0.19	0.08–0.47	<0.01	13.12
ACEIs & ARBs – in hospital	0.44	0.31–0.64	<0.01	18.66
Beta Blockers – in hospital	0.73	0.48–1.10	>0.13	2.31
Aspirin – in hospital	0.49	0.33–0.71	<0.01	13.87
Thrombolytics – in hospital	0.57	0.39–0.83	<0.01	8.51
Time Cohorts (MICH1)*				16.95**
MICH2	1.46	0.99–2.15	>0.05	3.57
HARP	0.16	0.05–0.46	<0.01	11.48

* Reference categories are in parentheses;

** χ^2^-difference value for all categories of variable;

*** Natural logarithm of Charlson Index

Model Information:

No. of In-hospital Deaths: 138; Model Likelihood Ratio χ^2 ^(df: 16) = 282.51;

Harrel's Concordance Statistic: 0.90

**Table 3:** Mortality Hazard After Discharge from Index Hospital by Patient Characteristics, Treatments and Time Cohort (n = 1,834)

**Variables:**	**Hazard Ratios**	**95% CI**	**p-value**	**Δχ**^2^**-value**
Age	1.03	1.01–1.04	<0.01	15.79
Gender (male)*	1.14	0.82–1.59	>0.45	0.57
Race (white)*	0.81	0.54–1.23	>0.32	0.98
Charlson Index***	1.81	1.33–2.46	<0.01	14.45
Ejection Fraction (<35%)*				6.78**
35%–44%	1.59	0.89–2.83	>0.11	2.49
≥45%	0.56	0.31–1.01	>0.05	3.71
missing	1.19	0.68–2.07	>0.54	0.37
Invasive Procedures (none)*				19.58**
CATH	0.56	0.34–0.91	<0.02	5.45
PCI	0.29	0.16–0.53	<0.01	15.75
CABG	0.39	0.22–0.67	<0.01	11.24
ACEIs & ARBs – discharge	1.06	0.75–1.48	>0.75	0.10
Beta Blockers – discharge	0.54	0.36–0.81	<0.01	8.78
Aspirin – discharge	0.79	0.56–1.10	>0.15	1.99
Time Cohorts (MICH1)*				1.67**
MICH2	1.29	0.87–1.92	>0.21	1.56
HARP	1.19	0.77–1.85	>0.43	0.62

* Reference categories are in parentheses;

** χ^2^-difference value for all categories of variable;

*** Natural logarithm of Charlson Index

Model Information:

No. of Post-Hospital Discharge Deaths: 152; Model Likelihood Ratio χ^2 ^(df: 15) = 203.73;

Harrel's Concordance Statistic: 0.81
